# Comparative Analysis of Cystamine and Cysteamine as Radioprotectors and Antioxidants: Insights from Monte Carlo Chemical Modeling under High Linear Energy Transfer Radiation and High Dose Rates

**DOI:** 10.3390/ijms251910490

**Published:** 2024-09-29

**Authors:** Samafou Penabeï, Jintana Meesungnoen, Jean-Paul Jay-Gerin

**Affiliations:** Department of Medical Imaging and Radiation Sciences, Faculty of Medicine and Health Sciences, Université de Sherbrooke, 3001, 12th Avenue Nord, Sherbrooke, QC J1H 5N4, Canada; samafou.penabei@usherbrooke.ca (S.P.); jintana.meesungnoen@usherbrooke.ca (J.M.)

**Keywords:** cystamine/cysteamine, radioprotector/antioxidant, linear energy transfer (LET), dose rate, ferrous sulfate (Fricke) dosimeter, radiolysis, Monte Carlo multi-track chemical modeling, radiation chemical yields (*G* values), FLASH radiotherapy

## Abstract

This study conducts a comparative analysis of cystamine (RSSR), a disulfide, and cysteamine (RSH), its thiol monomer, to evaluate their efficacy as radioprotectors and antioxidants under high linear energy transfer (LET) and high-dose-rate irradiation conditions. It examines their interactions with reactive primary species produced during the radiolysis of the aqueous ferrous sulfate (Fricke) dosimeter, offering insights into the mechanisms of radioprotection and highlighting their potential to enhance the therapeutic index of radiation therapy, particularly in advanced techniques like FLASH radiotherapy. Using Monte Carlo multi-track chemical modeling to simulate the radiolytic oxidation of ferrous to ferric ions in Fricke-cystamine and Fricke-cysteamine solutions, this study assesses the radioprotective and antioxidant properties of these compounds across a variety of irradiation conditions. Concentrations were varied in both aerated (oxygen-rich) and deaerated (hypoxic) environments, simulating conditions akin to healthy tissue and tumors. Both cystamine and cysteamine demonstrate radioprotective and strong antioxidant properties. However, their effectiveness varies significantly depending on the concentration employed, the conditions of irradiation, and whether or not environmental oxygen is present. Specifically, excluding potential in vivo toxicity, cysteamine substantially reduces the adverse effects of ionizing radiation under aerated, low-LET conditions at concentrations above ~1 mM. However, its efficacy is minimal in hypoxic environments, irrespective of the concentration used. Conversely, cystamine consistently offers robust protective effects in both oxygen-rich and oxygen-poor conditions. The distinct protective capacities of cysteamine and cystamine underscore cysteamine’s enhanced potential in radiotherapeutic settings aimed at safeguarding healthy tissues from radiation-induced damage while effectively targeting tumor tissues. This differential effectiveness emphasizes the need for personalized radioprotective strategies, tailored to the specific environmental conditions of the tissue involved. Implementing such approaches is crucial for optimizing therapeutic outcomes and minimizing collateral damage in cancer treatment.

## 1. Introduction

The ongoing rise in cancer incidence presents a formidable challenge to global health systems, with various types of malignancies responsible for millions of deaths each year. According to the American Cancer Society, the global incidence of cancer was 19.3 million new cases in 2020, with projections indicating an increase to 28.4 million by 2040 [1]. In this context, radiation therapy (RT) plays a pivotal role in cancer treatment, offering significant potential to control disease progression. Employed in approximately half of all cancer treatment regimens, RT underscores its essential contribution to both palliative care and curative treatments [2]. The effectiveness of RT hinges on its precision in targeting cancerous cells while sparing the surrounding healthy tissues. This delicate balance is essential for optimal tumor control and minimal damage to non-cancerous cells, underscoring the vital role of radioprotective agents in enhancing the efficacy and safety of cancer therapy [3,4,5]. These agents play a crucial role in protecting healthy cells from the harmful effects of radiation, substantially reducing the side effects associated with RT. Their impact on lowering patient morbidity and enhancing treatment outcomes is significant, as they greatly improve the quality of life for patients. Yet, the development of radioprotective strategies faces challenges, particularly in formulating agents that can precisely distinguish between cancerous and healthy tissues. Successfully incorporating these agents into clinical practice represents a key advancement, aiming to refine RT into a more targeted and manageable treatment [6,7].

The emergence of ultra-high-dose-rate irradiation techniques like FLASH-RT marks a revolutionary advance in radiation oncology (e.g., see [8,9,10,11,12]). FLASH-RT leverages an exceptional biological effect by delivering irradiation doses at rates significantly higher than conventional methods, minimizing damage to healthy tissues while efficiently targeting tumor cells. Early research on FLASH-RT suggests potential for reducing side effects, heralding a new era in oncology where treatments are not only effective, but also less burdensome for patients. However, integrating FLASH-RT and other advanced technologies into routine clinical practice poses significant challenges, requiring thorough research to ensure their effectiveness across various cancer types and patient groups. The improvement these technologies offer in sparing healthy tissues more effectively could profoundly transform the landscape of cancer management [13,14,15].

Furthermore, the pivotal role of complementary strategies, such as radioprotective agents, is paramount for fully harnessing the therapeutic potential of radiotherapy. The ongoing evolution of radiotherapy, marked by the exploration of higher dose rates and enhanced radiation protection, reflects the dynamic and evolving nature of cancer treatment strategies. As the scientific community delves deeper into the capabilities and limitations of FLASH-RT, the critical need for sustained research into radiation protection becomes increasingly clear.

The development of non-toxic, effective radioprotective compounds is crucial for enhancing the efficacy of advanced radiation therapy techniques like FLASH-RT. These compounds are designed not only to enhance therapeutic outcomes by mitigating the adverse effects of high-dose radiation, but also to act as vital protective measures against unintended radiation exposures in clinical settings, during accidents, and in specific, occupational hazards. This dual functionality underscores their pivotal role in both enhancing medical treatments and ensuring radiation safety, thereby reinforcing their importance in modern medical practice [7,16,17,18,19].

### 1.1. Radiolysis of Water: Effects of Radiation Quality and Dose Rate on the Formation of Radical and Molecular Products

Living cells and tissues, predominantly composed of water, make the study of radiation chemistry in aqueous solutions essential for understanding the initial stages of the complex series of radiobiological events triggered by ionizing radiation [20,21]. In this context, the primary reactive species produced during the radiolytic decomposition of aerated pure water include the hydrated electron (e^−^_aq_), hydrogen atom (H^•^), molecular hydrogen (H_2_), hydroxyl radical (^•^OH), hydrogen peroxide (H_2_O_2_), hydronium ion (H_3_O^+^), hydroxide ion (OH−), and the hydroperoxyl/superoxide anion radicals (HO_2_^•^/O_2_^•−^, depending on pH) [22,23,24,25]. Among these, ^•^OH, e^−^_aq_, and H_3_O^+^ are produced in the highest concentrations [26]. The hydroxyl radical is especially critical due to its high reactivity and its potential to readily interact with DNA, causing structural changes [20]. These radiolytic species, including those often referred to as radiation-induced ‘reactive oxygen species’ (ROS)—such as, e.g., peroxynitrite (ONOO^−^) formed from the reaction of nitrite oxide ^•^NO with superoxide anions, peroxyl (RO_2_^•^) and alkoxyl (RO^•^) radicals, singlet oxygen (^1^O_2_), etc.—play a significant role in chemical modifications within cellular environments. These species can initiate signaling pathways or damaging effects, potentially leading to observable biological responses [27].

The behavior of these species is strongly influenced by the quality of radiation, or equivalently, by its linear energy transfer (LET), which measures the energy deposited per unit path length as radiation travels through the medium [28]. Low-LET radiation, also known as sparsely ionizing radiation—including γ-rays from ^60^Co or ^137^Cs, fast electrons, or high-energy protons or charged particles—typically induces localized energy-loss events within small, more or less spherical volumes known as “spurs” [29,30]. These spurs are sufficiently spaced to allow the reactive species within them to engage in individual chemical pathways without significant interactions with neighboring spurs [31]. In contrast, high-LET radiation, such as low-energy protons or heavy ions, neutrons, or α-particles, generates densely packed, continuous ionizing tracks of a cylindrical shape, resulting in a concentrated and overlapping distribution of reactive species. This close proximity fosters radical–radical interactions, enhancing combination and recombination reactions in the diffusing tracks, thus reducing the yield of free radicals while promoting the formation of stable molecular entities like H_2_ and H_2_O_2_. The dense spatial arrangement of these radiation tracks under high-LET conditions markedly alters the radiolytic product profile compared to that seen with low-LET radiation [32,33].

Under standard irradiation conditions with no dose-rate effects, radiation tracks do not overlap, and the chemical effects of irradiation can be summed as the separate effects of individual tracks, each evolving independently over time. However, increased radiation dose rates alter the physicochemical and spatiotemporal dynamics significantly due to overlapping radiation tracks, leading to enhanced inter-track chemistry. This scenario mirrors high-LET conditions [34], characterized by higher ionization density, where the yield of free radicals decreases while that of molecular products increases across the developing radiation tracks. As such, elevated dose rates associated with high-LET conditions significantly impact the radiolysis, intensifying interactions among radiation tracks and altering the usual chemical yields and mechanisms observed at lower dose rates (e.g., see [35,36] and references cited therein). This intricate interplay of chemical reactions under diverse radiolytic conditions underscores the complex nature of radiation interactions with water, necessitating a comprehensive understanding for applications like radiotherapy and nuclear reactor chemistry, where precise control over radiation effects is paramount.

### 1.2. Assessing the Radioprotective, Free-Radical Scavenging, and Antioxidant Properties of Cystamine and Cysteamine Using the Fricke Dosimeter: Influence of Proton Energy Variability and Dose Rate

Within the intricate cellular framework, radioprotective agents are pivotal in regulating defense mechanisms against radiation-induced harm. These agents are characterized by their multifaceted mechanisms of action, including free-radical scavenging and hydrogen atom donation [17,20]. These capabilities are crucial for mitigating damage caused by reactive intermediates that arise during water radiolysis, often referred to as the ‘indirect effect’ in radiobiology [4]. Importantly, these reactive species are efficiently intercepted—or scavenged—by these agents before they can interact with critical cellular components, especially DNA [3,37].

The role of compounds with sulfhydryl (–SH) groups in radiation protection has long been recognized [38,39,40,41]. These compounds are valued for their cytoprotective properties, mainly attributed to their ability to donate labile hydrogen atoms. This action effectively mitigates both direct and indirect molecular damage to DNA and other essential biomolecules [4,20,42]. By competing with molecular oxygen—a diradical—for reaction sites on critical DNA radicals, these compounds inhibit the formation of peroxyl radicals and prevent the permanent fixation of damage [43]. This strategic intervention not only shields DNA and critical cellular structures from damage, thereby ensuring genomic integrity, but also supports cellular repair mechanisms, significantly enhancing cell survival after radiation exposure.

Cystamine, a diamino-disulfide compound (RSSR, where R = NH_2_–CH_2_–CH_2_), and cysteamine, its reduced aminothiol form (RSH), are both derivatives of the amino acid cysteine (HS–CH_2_–CH(NH_2_)–COOH), a common building block of most proteins. Recognized for their radioprotective qualities, these compounds are effective in mitigating oxidative stress in cells and tissues. In cellular reducing environments, cystamine rapidly converts into two cysteamine molecules through the cleavage of its disulfide bond [44,45]. Research indicates that cysteamine is the active metabolite responsible for cystamine’s radioprotective effects in vivo [46,47]. The coexistence of these compounds in a redox equilibrium within cells underscores the intricate interplay between their metabolism and biological functions. Moreover, beyond their capacity as free-radical scavengers, both compounds exhibit neuroprotective properties in experimental animal models and some clinical trials, positioning them as potential treatments for neurodegenerative disorders like Parkinson’s and Huntington’s diseases [48]. Additionally, they have shown antiviral efficacy against influenza A, hepatitis A, and the human immunodeficiency virus HIV-1, expanding their therapeutic potential [49].

Several prior studies [50,51], including our own [37,52,53,54,55], have employed the well-established radiolytic oxidation of ferrous (Fe^2+^) to ferric (Fe^3+^) ions in aqueous ferrous (Fricke) dosimeter solutions [23,56,57] to quantify the radical-scavenging properties of cystamine and other notable disulfide compounds, such as cystine (the oxidized dimer of cysteine linked by a disulfide bond) and oxidized glutathione (GSSG). Originally developed for dose measurement, the Fricke dosimeter has also proven to be, at the molecular level, a most valuable tool for investigating the impact of scavengers on primary chemical species produced during water radiolysis, particularly the radiolytic yield of ferric ions, denoted as *G*(Fe^3+^) (e.g., see [58,59,60,61]). When radioprotective agents like cystamine or cysteamine are present in an irradiated Fricke solution, they competitively react with the products of water radiolysis before these can oxidize Fe^2+^ ions. This results in a reduced *G*(Fe^3+^) value, effectively preserving the Fe^2+^ ions. The observed reduction in *G*(Fe^3+^) in the presence of cystamine [50,51,53] was further substantiated through Monte Carlo simulations of the radiolysis of Fricke-cystamine solutions under both aerated and deaerated conditions [53,54].

Our recent work has focused on elucidating the fundamental molecular mechanisms underlying the radioprotective and antioxidant actions of cystamine. We employed our IONLYS-IRT Monte Carlo track chemistry computer code (see Meesungnoen and Jay-Gerin [33] and cited references) to simulate the radiolysis of Fricke-cystamine solutions under 300 MeV proton irradiation, mimicking the low-LET conditions characteristic of Compton electrons (in the MeV range) released following the absorption of ^60^Co γ rays in liquid water. With a solid understanding of cystamine’s radiation chemistry, we successfully reproduced the experimental yields of Fe^3+^ across a wide cystamine concentration range from 10⁻^6^ to 1 M [53]. Moreover, we assessed the impact of radiation’s LET on cystamine’s radioprotective efficacy by adjusting the energy of the irradiating protons from 150 keV to 500 MeV, corresponding to LET values from ~72 keV/μm to 0.23 keV/μm [37]. Our results indicated that cystamine’s protective effect decreased with increasing LET. We also explored the influence of dose rate on *G*(Fe^3^⁺), particularly under high-dose-rate conditions akin to those used in advanced radiotherapeutic techniques like FLASH-RT. Employing a multi-track irradiation model along with an advanced version of our IONLYS-IRT Monte Carlo code [34], we simulated the exposure of Fricke-cystamine solutions to single, instantaneous pulses of 300 MeV protons [54]. Findings showed that cystamine provides superior radioprotection under pulsed high-dose-rate conditions compared to continuous, low-dose-rate irradiations. Finally, we delved into cystamine’s radioprotective activity against fast carbon ion (^12^C^6+^ nuclei, i.e., carbon atoms stripped from all of their electrons) irradiation, examining a broad energy range from 6 to 500 MeV per nucleon, aligning with LET values from ~250 keV/μm to 9.3 keV/μm [55]. This range covers energies typically used in clinical carbon ion hadrontherapy, notably around 400 MeV per nucleon. Our simulations shed light on the optimal conditions for cystamine’s radioprotective action, emphasizing the significant influence of LET, particularly the very high LETs typical of the “Bragg peak” region at the ion’s range terminus [62], where we noted a marked reduction in cystamine’s effectiveness, highlighting the need for a careful consideration of LET when formulating radioprotective strategies.

Building on our previous research on cystamine, the present study aims to explore the radioprotective and antioxidant properties of its reduced form, cysteamine. We employ the same innovative Monte Carlo-based approach, previously described [33,34,53,54,55], alongside the radiolytic oxidation of Fe^2+^ to Fe^3+^ ions in the Fricke chemical dosimeter. This methodology allows us to assess cysteamine’s radical-scavenging capabilities under various proton irradiation conditions. Thus, we establish the groundwork for a comparative analysis of cysteamine and cystamine, focusing on their respective protective mechanisms in both aerated and deaerated environments.

We aim to assess the impact of a broad energy range of proton irradiation (from 0.15 to 300 MeV, corresponding to LET values from ~72 to 0.3 keV/μm) under high dose rates using 300 MeV protons, a situation relevant to FLASH-RT. Additionally, we will explore a wide range of cysteamine concentrations (from 10^−6^ to 0.1 M). Our research significantly advances the scientific understanding of the differential capacity of cystamine versus cysteamine to influence redox reactions under various irradiation scenarios. By examining the extensive range of cysteamine concentrations, we elucidate the precise mechanisms through which this compound modulates radiolytic reactions, particularly in environments with varying oxygen levels.

The integration of our upgraded IONLYS-IRT Monte Carlo code, coupled with a comprehensive multi-track irradiation framework [34], represents a significant enhancement in predictive and analytical methodologies. This advancement not only improves our ability to assess the chemical kinetics of radiolytic interactions but also substantially contributes to refining radioprotective measures. These improvements are crucial for both therapeutic interventions, such as the “FLASH effect” in radiobiology and emergency nuclear safety protocols. This research offers potential applications in enhancing the safety and efficacy of radiation-based treatments and emergency response strategies.

Throughout this article, radiation chemical yields are expressed in the standard units of molecules formed (or consumed) per 100 eV of energy absorbed, denoted as *g*(X) for primary (or “escape”) yields and *G*(X) for yields derived from experimental observations [23,25]. To align with the System International of Units (SI), we use the conversion: 1 molecule/100 eV ≈ 0.10364 μmol/J [24].

## 2. Results and Discussion

### 2.1. Kinetics of Fe^3+^ Formation in Aerated and Deaerated Fricke Solutions Exposed to 0.15–300 MeV (~72–0.3 keV/μm) Proton Irradiation without Added Cystamine or Cysteamine, in the Absence of Dose-Rate Effects

**Figure 1:**
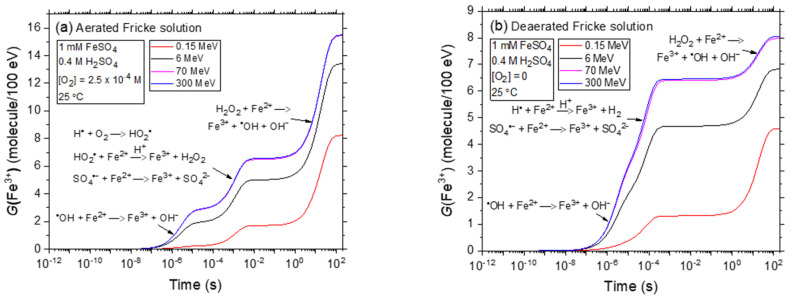
Time evolution of *G*(Fe^3+^) for incident proton energies of 300, 70, 6, and 0.15 MeV in the radiolysis of aerated (Panel (**a**)) and deaerated (Panel (**b**)) Fricke solutions at 25 °C, over the interval of 1 ps–200 s. These data are for no dose-rate effects (*N* = 1). In solutions containing 0.4 M H_2_SO_4_, a minor fraction of ^•^OH radicals reacts with HSO_4_^−^ to form the sulfate radical SO_4_^•−^ (Reaction (4)). Despite this reaction, the overall yield of Fe^3+^ remains consistent with the predictions of Equations (10) and (11), SO_4_^•−^ reacting with Fe^2+^ in a manner analogous to that of ^•^OH (Reaction (5)) [53].

Figure 1a,b displays the kinetics of Fe^3+^ formation in the radiolysis of the Fricke solution under both aerated and deaerated conditions, respectively, based on our simulations. These simulations were conducted at 25 °C, using proton energies of 0.15 MeV (LET~72 keV/μm), 6 MeV (LET~6.8 keV/μm), 70 MeV (LET~0.9 keV/μm), and 300 MeV (LET~0.3 keV/μm) over 1 ps to 200 s, without considering dose-rate effects (*N* = 1). The time-dependent *G*(Fe^3+^) reflects reactions of Fe^2+^ with radiolysis products like ^•^OH, HO_2_^•^ in oxygen-rich environments, and H^•^ in oxygen-depleted conditions, along with SO_4_^•−^ and H_2_O_2_. Notably, our computed values of *G*(Fe^3+^) for 300 MeV proton irradiation closely match the established values for ^60^Co γ-ray or fast electron irradiation, which are 15.5 ± 0.2 and 8.2 ± 0.3 molecules/100 eV in the presence and absence of O_2_, respectively [23,57,63,64,65,66,67].

As outlined in Section 3.1, the pronounced decline in *G*(Fe^3+^) values from low-LET 300 MeV to high-LET 0.15 MeV irradiating protons is largely attributed to variations in the spatial distribution of energy deposition. The transition from sparse to densely ionizing radiation amplifies the relevance of intra-track and radical–radical combination reactions that yield molecular products. According to Equations (10) and (11), the lower the escape rate of free radicals from radiation tracks, the lower the Fricke *G* values. Specifically, when shifting from protons at 300 MeV (LET~0.3 keV/μm) to 0.15 MeV (LET~72 keV/μm) the *G*(Fe^3+^) value reduces from ~15.5 to about 8.25 molecules per 100 eV in the presence of O_2_, and from ~8.05 to 4.6 molecules per 100 eV in the absence of O_2_. These results are consistent with the many experimental data cited in the literature regarding the impact of LET on the chemical responses and yields of the Fricke dosimeter [23,37,55,66,67,68,69,70,71,72,73,74].

### 2.2. Comparison of Fe^3+^ Ion Yields in Aerated and Deaerated Fricke-Cystamine and Fricke-Cysteamine Solutions Exposed to 0.15–300 MeV (~72–0.3 keV/μm) Proton Irradiation, in the Absence of Dose-Rate Effects

**Figure 2:**
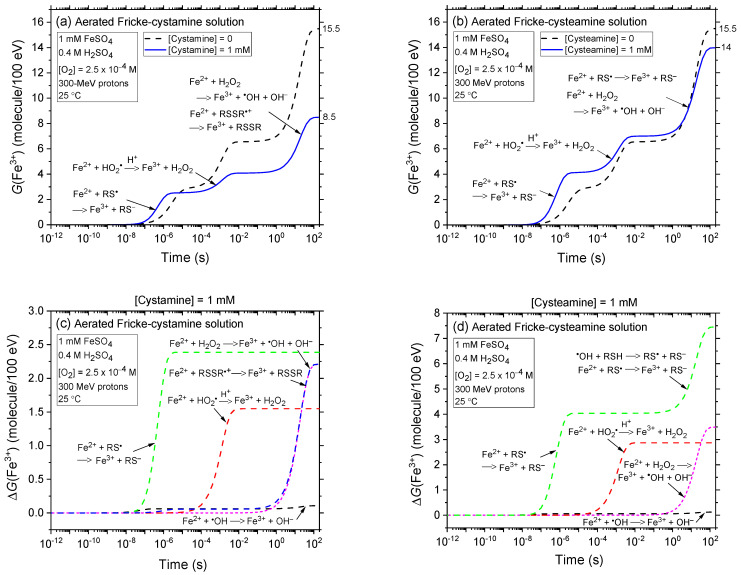

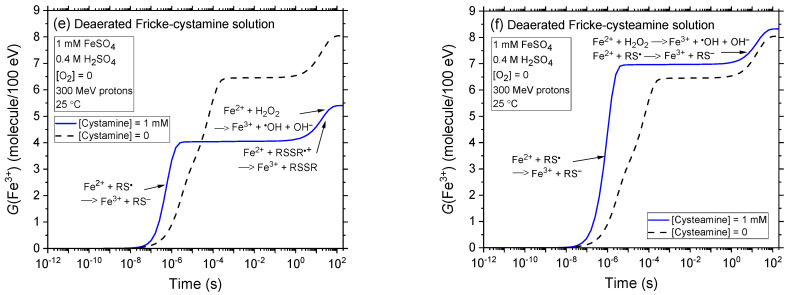
Panels (**a**,**b**) display the temporal evolution of *G*(Fe^3+^) during the radiolysis of aerated Fricke solutions mixed with 1 mM of either cystamine or cysteamine, respectively, when exposed to 300 MeV proton irradiation, over the interval from 1 ps to 200 s. The solid blue lines in these panels represent our simulations of the kinetics of Fe^3+^ ion formation for each scenario. For comparison, the dashed black lines show the time evolution of *G*(Fe^3+^) in the radiolysis of standard aerated Fricke solutions without the addition of cystamine or cysteamine. Panels (**c**,**d**) highlight the time dependence of the extents Δ*G*(Fe^3+^), measured in molecules per 100 eV, of the various reactions that contribute to the formation of Fe^3+^ ions under conditions analogous to those in Panels (**a**,**b**). Finally, Panels (**e**,**f**) display the temporal evolution of *G*(Fe^3+^) during the radiolysis of deaerated Fricke solutions containing 1 mM of either cystamine or cysteamine, respectively, when exposed to 300 MeV proton irradiation, over the same time interval from 1 ps to 200 s. The solid blue lines here also represent our simulations of the kinetics of Fe^3+^ ion formation, while the dashed black lines provide a comparison with the evolution of *G*(Fe^3+^) in deaerated Fricke solutions without these additives. All of the data are at 25 °C, for no dose-rate effects (*N* = 1).

In Figure 2a,b, we examine the effects of adding 1 mM cystamine (RSSR) and cysteamine (RSH) on the kinetics of Fe^3+^ formation, based on our simulations of the radiolysis of the Fricke dosimeter using 300 MeV protons (~0.3 keV/μm) in aerated conditions. The findings demonstrate that in both scenarios, the oxidation of Fe^2+^ to Fe^3+^ predominantly occurs through reactions with HO_2_^•^, H_2_O_2_, and the radical species RS^•^ and RSSR^•+^. Notably, reaction (27), which involves Fe^2+^ ions and thiyl radicals RS^•^, proceeds most rapidly, completing within just a few microseconds. This is clearly indicated in Figure 2c,d, where we display the time profiles for the extent Δ*G*(Fe^3+^) of each reaction contributing to Fe^3+^ formation. Interestingly, the thiyl radicals primarily originate from reaction (13) in the case of cystamine and from reactions (19) and (20) for cysteamine. H^•^ atoms that do not react with cystamine or cysteamine subsequently react with O_2_ via reaction (2), forming HO_2_^•^. This species then oxidizes Fe^2+^ to Fe^3+^. However, the oxidation process by HO_2_^•^ is comparatively slower, taking ~10 ms to complete. Finally, beyond ~1 s, two sets of reactions predominantly contribute to the formation of Fe^3+^. Initially, reaction (8) occurs, followed closely by reactions (14) and (28) in the case of cystamine, and reactions (20) and (27) for cysteamine, all of which conclude by about 200 s. These latter reactions are closely interconnected; the production of H_2_O_2_ in reaction (8) not only contributes directly to the Fe^3+^ yield but also facilitates the formation of a stoichiometrically-equivalent yield of ^•^OH radicals. In the scenarios being examined—solutions containing 1 mM Fe^2+^ ions along with 1 mM cystamine or cysteamine—almost all of the ^•^OH radicals formed are immediately scavenged by either cystamine or cysteamine before they can react with Fe^2+^. At these equal concentrations, the competition for ^•^OH radicals heavily favours the scavenging by cystamine or cysteamine over their reaction with Fe^2+^. This is clearly illustrated by the rate constants: both reactions, (14) and (20), have a rate constant of 1.7 × 10^10^ M^−1^ s^−1^, which is ~45 times higher than that for reaction (3), at 3.8 × 10^8^ M^−1^ s^−1^. Additionally, the fact that ^•^OH radicals are preferentially scavenged by cystamine or cysteamine not only eliminates the contribution of reaction (3) to the formation of Fe^3+^ but also prevents the occurrence of reaction (5). This is due to the absence of reaction (4), which would normally lead to the production of SO_4_^•−^ radicals, thus stopping the chain of reactions that follow.

In Figure 2e,f, we present the kinetics of Fe^3+^ formation in the radiolysis of the Fricke solution containing 1 mM of either cystamine or cysteamine, under deaerated conditions, using our simulations with 300 MeV proton irradiation. The results are largely similar to those shown in Figure 2a,b, with a notable exception: under deaerated conditions, reaction (2), where H^•^ atoms react with O_2_, does not occur.

The data presented in Figure 2a,b clearly show that, under 300 MeV proton irradiation (low-LET radiation), the *G*(Fe^3+^) value at ~200 s is reduced in the presence of 1 mM cystamine or cysteamine. This decrease is substantially more pronounced with cystamine, where *G*(Fe^3+^) falls from ~15.5 to about 8.5 molecules per 100 eV—a reduction of ~7 *G*-units—compared to the Fricke solution without added cystamine, as depicted in Figure 2a. In contrast, with cysteamine, the decrease in *G*(Fe^3+^) is much less significant, dropping from ~15.5 to 14 molecules per 100 eV (a ~1.5 *G*-unit decrease; Figure 2b). These findings confirm that cystamine and cysteamine are effective at scavenging the primary products of acid water radiolysis, specifically H^•^ atoms and ^•^OH radicals, as outlined in reactions (13), (14), (19), and (20). This scavenging action is pivotal in preventing the radiolytic oxidation of ferrous ions in irradiated Fricke solutions. Mechanistically, the radical-capturing capabilities of cystamine and cysteamine shape their “antioxidant profiles”, allowing them to compete with Fe^2+^ ions for the free radicals produced during water irradiation. We note, however, at a concentration of 1 mM, cystamine exhibits markedly higher radioprotective efficacy than cysteamine. This difference in radioprotective effectiveness between cystamine and cysteamine is also evident in irradiated deaerated solutions, with cystamine demonstrating superior radioprotective properties, while cysteamine exhibits no efficacy, as illustrated in Figure 2e,f. We will explore this discrepancy in detail in Section 2.3. As we will discuss, the difference in effectiveness depends significantly on the concentration and the presence or absence of O_2_ at a given LET.

**Figure 3:**
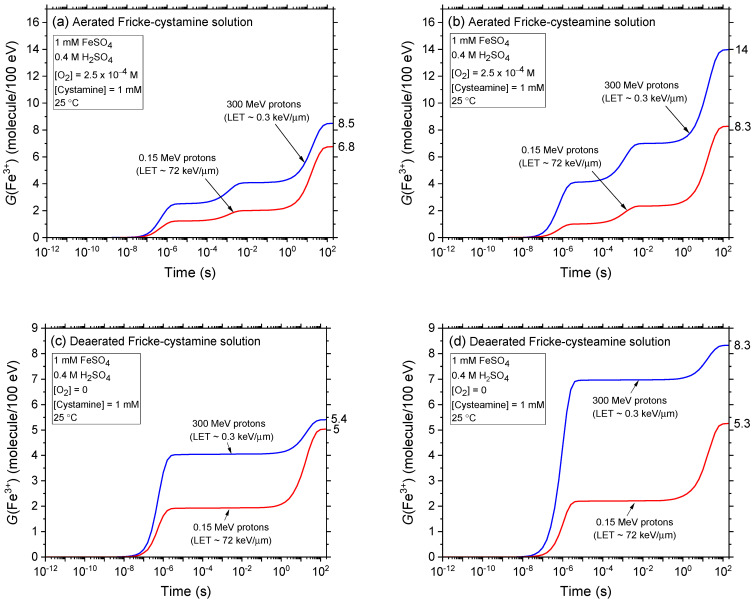
Time evolution of *G*(Fe^3+^) during the radiolysis of Fricke solutions, both aerated (Panels (**a**,**b**)) and deaerated (Panels (**c**,**d**)) containing 1 mM of either cystamine or cysteamine, when exposed to proton irradiation at energies of 300 MeV (~0.3 keV/μm; depicted by the blue lines) and 0.15 MeV (~72 keV/μm; depicted by the red lines), over the interval from 1 ps to 200 s. For the aerated solutions, simulations were conducted using a dissolved O_2_ concentration of 0.25 mM at 25 °C. As outlined in Figure 2a–f, the oxidation of Fe^2+^ to Fe^3+^ primarily occurs through reactions with HO_2_^•^, H_2_O_2_, and the radical species RS^•^ and RSSR^•+^. The data presented here do not account for dose-rate effects (*N* = 1).

Figure 3a–c illustrates the impact of LET on the kinetics of Fe^3+^ ion formation during the radiolysis of Fricke solutions containing 1 mM of either cystamine or cysteamine, under aerated and deaerated conditions. Our simulations employed low-LET 300 MeV and high-LET 0.15 MeV irradiating protons. As depicted in Figure 3a,b, under aerated conditions, the *G*(Fe^3+^) value at ~200 s post-irradiation drops from ~8.5 to 6.8 molecules per 100 eV (a 1.7 *G*-unit decrease) with cystamine when LET increases from ~0.3 to 72 keV/μm. With cysteamine, the decrease is more pronounced, from ~14 to 8.3 molecules per 100 eV (a 5.7 *G*-unit drop). Figure 3c,d reveals similar trends under deaerated conditions. As discussed in Section 3.1, these observations are attributed to the enhanced role of intra-track processes in denser ionizing radiations, which limit the escape of free radicals from the radiation tracks, thereby reducing their availability to oxidize Fe^2+^ ions [23,66,73,74]. Importantly, LET significantly affects the radioprotection of Fe^2+^ ions in the radiolysis of Fricke-cystamine and Fricke-cysteamine solutions. However, at a concentration of 1 mM, LET is markedly less effective in reducing *G*(Fe^3+^) when cystamine is used as opposed to cysteamine, regardless of the oxygenation conditions.

### 2.3. Impact of Cystamine and Cysteamine Concentrations on Fricke Yield across Proton Energies from 300 to 0.15 MeV without Dose-Rate Effects

**Figure 4:**
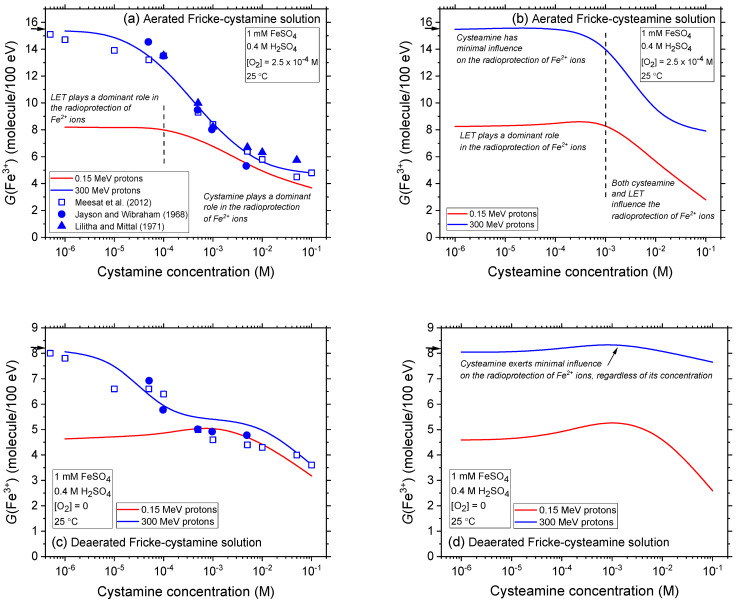
Dependence of Fe^3+^ ion yield from our simulations of radiolysis of Fricke-cystamine and Fricke-cysteamine solutions, at ~200 s after ionization, on cystamine and cysteamine concentrations from 10^−6^ to 0.1 M. We used 300 MeV (LET ~ 0.3 keV/μm) and 0.15 MeV (LET ~ 72 keV/μm) irradiating protons, in the absence of dose-rate effects (*N* = 1). Results are shown under both aerated (Panels (**a**,**b**)) and deaerated (Panels (**c**,**d**)) conditions at 25 °C. Experimental data, highlighted in blue in Panel (**a**), are referenced from [50,51,53]. Vertical dashed lines in Panels (**a**,**b**) at concentrations of ~10^−4^ M cystamine and ~1 mM cysteamine, respectively, mark transitions between distinct radioprotective mechanisms (see text). Below these concentrations, *G*(Fe^3+^) levels remain nearly constant, influenced primarily by LET. Above these thresholds, effects are predominantly driven by cystamine in Panel (**a**) and a synergy of LET and cysteamine in Panel (**b**). Notably, in the low-LET 300 MeV proton radiolysis in deaerated Fricke-cysteamine (Panel (**d**)), *G*(Fe^3+^) remains relatively stable across all concentrations studied. Arrows next to the figures show the accepted yields (15.5 ± 0.2 and 8.2 ± 0.3 molecules per 100 eV, respectively) for the aerated and deaerated Fricke dosimeter when exposed to ^60^Co γ-rays or fast electrons, without any additives.

The influence of cystamine and cysteamine concentrations on Fricke yield is depicted in Figure 4a–d. This figure compares our calculated *G*(Fe^3+^) values for Fricke-cystamine and Fricke-cysteamine solutions, both with and without oxygen, when irradiated by protons at energies of 300 and 0.15 MeV. These values are reported for concentrations ranging from 10^−6^ to 0.1 M. Overall, adding cystamine and cysteamine significantly reduces *G*(Fe^3+^) in both aerated and deaerated conditions. This reduction, previously detailed for cystamine in [37,50,51,52,53,54,55], underscores the strong radical-scavenging capabilities of these compounds, especially under low-LET proton irradiation. They operate by competing with Fe^2+^ ions for free radicals produced during water radiolysis, demonstrating their efficacy as radioprotectors and potent antioxidants. Further details and implications of these findings are discussed below.

Remarkably, Figure 4a,c demonstrates that our calculated Fe^3+^ yields from low-LET 300 MeV (~0.3 keV/μm) incident protons accurately reproduce, without any adjustable parameters, the experimentally measured yields of Fe^3+^ for aerated and deaerated Fricke-cystamine solutions irradiated by X-rays and ^60^Co γ-rays, as reported in [50,51,53]. This precise alignment between calculated and measured *G*(Fe^3+^) values at low LET strongly supports the accuracy and reliability of the chemical reaction scheme employed in this study for describing the radiation chemistry of cystamine and, by extension, cysteamine in these solutions.

The data displayed in Figure 4a,b, which examine the radiolysis of Fricke-cystamine and Fricke-cysteamine solutions by both low-LET 300 MeV and high-LET 0.15 MeV irradiating protons in an aerated environment, leads to several insightful observations. Notably, the figure distinctly shows that the observed reduction in *G*(Fe^3+^) results from two synergistic radioprotective mechanisms: one stemming from the intrinsic influence of LET itself, i.e., the physical properties of the radiation, and the other driven by the chemical activity of cystamine or cysteamine in the solutions.

Specifically, at concentrations below ~10^−4^ M for cystamine and below 1 mM for cysteamine, whether under low- or high-LET irradiation, these compounds exhibit limited efficacy in reducing the Fricke yield. For instance, at the lowest proton energy of 0.15 MeV (corresponding to ~72 keV/μm, our highest LET), *G*(Fe^3+^) remains relatively constant, indicating minimal dependence on the concentrations of cystamine or cysteamine. As explained in Section 3.1, this behavior is attributed to higher LET levels reducing radical formation—the targets for cystamine/cysteamine interaction—while simultaneously increasing the production of molecular entities such as H_2_O_2_, H_2_, and reformed water. These entities are largely unreactive with both cystamine or cysteamine. Furthermore, it is important to note that cysteamine, unlike cystamine, has virtually no effect on the radioprotection of Fe^2+^ ions when exposed to low-LET 300 MeV irradiating protons, even at concentrations as high as 1 mM. Specifically, at this concentration, *G*(Fe^3+^) is reduced to ~8.4 molecules per 100 eV (a decrease of ~7.1 *G*-units) in the presence of cystamine, whereas it remains at ~13.9 molecules per 100 eV (a decrease of only ~1.6 *G*-units) with cysteamine. Hence, in this low concentration range up to about 1 mM, cystamine is significantly more effective as a radioprotector than cysteamine. At higher concentrations, still within low LET conditions, both cystamine and cysteamine demonstrate substantial radioprotective properties. However, cystamine remains superior, reducing *G*(Fe^3+^) to 4.8 molecules per 100 eV at a concentration of 0.1 M, whereas with cysteamine, it remains at 7.9 molecules per 100 eV.

A noteworthy observation emerges when cysteamine is used under high-LET radiation conditions. Many studies indicate that chemical radioprotectors, particularly radioprotective aminothiols, are generally more effective against low-LET radiation. However, their efficacy significantly diminishes under high-LET radiation (see, e.g., [4,16,17,39,75]). Our results underscore this trend for cystamine; at 0.15 MeV proton irradiation and high concentrations, the reduction in *G*(Fe^3+^) is relatively modest and aligns with the reduction observed at low LET for the same concentration range (Figure 4a). In contrast, cysteamine exhibits quite a distinct behavior at concentrations exceeding 1 mM, where *G*(Fe^3+^) shows a substantial decrease. This demonstrates that the radioprotection of Fe^2+^ ions by cysteamine is not only influenced by LET, but also markedly by its distinct chemical properties. For example, at a concentration of 0.1 M—the highest concentration we examined—*G*(Fe^3+^) is reduced to 3.7 molecules per 100 eV with cystamine, compared to a further decrease to 2.8 molecules per 100 eV with cysteamine. This distinctive response suggests that cysteamine might offer more benefits than cystamine in specific medical applications like proton therapy or hadrontherapy. However, the effectiveness of cysteamine, much more so than cystamine, relies heavily on the use of relatively high concentrations. Yet, these elevated concentrations, while potentially more effective, could significantly restrict its clinical utility as a radioprotector due to possible in vivo toxicity or side effects, as discussed in recent literature [76].

Our examination of how cystamine and cysteamine concentrations influence Fricke yield values in deaerated conditions, as presented in Figure 4c,d, reveals results that generally align with those observed under aerated conditions. As anticipated, the reductions in *G*(Fe^3+^) are less pronounced in deaerated solutions, highlighting the Fricke dosimeter’s sensitivity to the oxygenation level of its environment, which can significantly affect its effectiveness. A particularly striking finding from this study is cysteamine’s unique behavior under low LET conditions: *G*(Fe^3+^) remains practically unchanged across all examined concentrations. This is in sharp contrast to the substantial decrease in *G*(Fe^3+^) noted in aerated solutions at concentrations above 1 mM, indicating that cysteamine has a negligible effect on the radioprotection of Fe^2+^ ions in the absence of O_2_, irrespective of concentration. A crucial implication of this finding is that cysteamine could serve as an effective radioprotector for healthy (oxygenated) tissue during radiotherapy, yet it offers no protection for hypoxic (oxygen-depleted) tumor tissue.

Summarizing these findings, it becomes apparent that the radioprotective effects of cysteamine compared to cystamine are multifaceted and vary with environmental conditions. Both compounds offer protection against the adverse effects of ionizing radiation, especially in oxygen-rich settings akin to healthy tissues. However, cystamine’s protective effectiveness significantly diminishes in low-oxygen, tumor-like environments, revealing a complex relationship between its radioprotective mechanisms and environmental oxygen levels. In contrast, cysteamine, though effective in oxygenated conditions, fails to provide comparable protection in deaerated environments.

**Figure 5:**
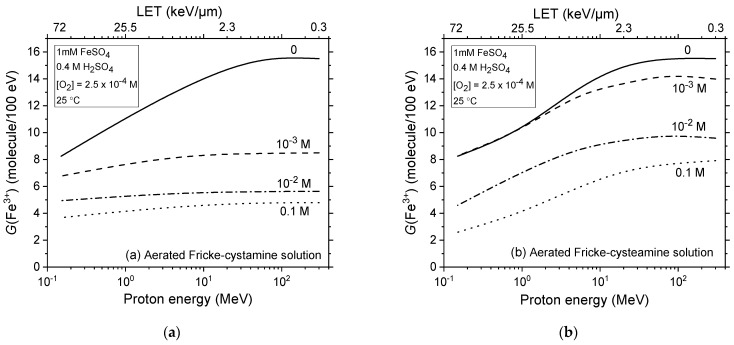
Effect of LET on *G*(Fe^3+^) values from Monte Carlo simulations of radiolysis in aerated Fricke-cystamine (Panel (**a**)) and Fricke-cysteamine (Panel (**b**)) solutions, using irradiating protons with energies ranging from 300 to 0.15 MeV, at a temperature of 25 °C, with a dissolved oxygen concentration of 2.5 × 10^−4^ M, and in the absence of dose-rate effects (*N* = 1). The lines indicate different concentrations of added cystamine or cysteamine: 1 mM (dashed line), 0.01 M (dash-dot line), and 0.1 M (dotted line). Solid lines represent results from the Fricke dosimeter without any additives, under the same irradiation conditions.

Figure 5a,b expands on the previous analysis by examining the impact of LET on the yield of ferric ions during the proton radiolysis of aerated Fricke-cystamine and Fricke-cysteamine solutions, using irradiating protons with initial energies from 300 to 0.15 MeV (or, equivalently, for LET values between ~0.3 and 72 keV/μm). It includes data at various concentrations of added cystamine or cysteamine, up to 0.1 M, offering a detailed and comprehensive overview.

Notably, the figure illustrates that cystamine provides superior radioprotection compared to cysteamine at concentrations of 1 mM and above, especially under low-LET conditions and throughout most of the LET range considered. However, at high LET levels, the radioprotective efficacy of cysteamine not only matches but can surpass that of cystamine, particularly at the highest concentration examined in this study, 0.1 M.

### 2.4. Comparison of Fe^3+^ Ion Yields in Aerated and Deaerated Fricke-Cystamine and Fricke-Cysteamine Solutions Exposed to 300 MeV (~0.3 keV/μm) Proton Irradiation, in the Presence of Dose-Rate Effects

**Figure 6:**
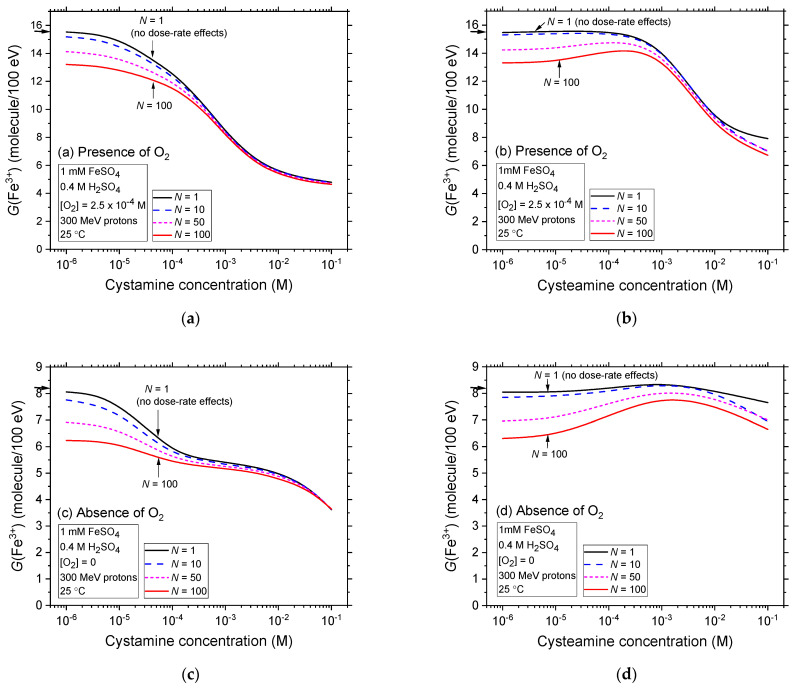
Effect of dose rate on the variation in Fe^3+^ ion yield obtained from our Monte Carlo multi-track chemistry simulations of the radiolysis of Fricke-cystamine and Fricke-cysteamine solutions, ~200 s post-irradiation, on cystamine and cysteamine concentrations ranging from 10^−6^ to 0.1 M. The simulations were conducted using single and instantaneous pulses of *N* 300 MeV (LET ~ 0.3 keV/μm) protons at 25 °C. Dose rate is denoted by *N*, representing the number of proton tracks per pulse. Data are presented under both aerated (Panels (**a**,**b**)) and deaerated (Panels (**c**,**d**)) conditions. The values of *N* chosen to demonstrate dose-rate effects include one (corresponding to the limit of one single-proton irradiation, which mimics ^60^Co γ-rays or fast-electron irradiation), ten, fifty, and one hundred. Arrows next to the panels indicate the accepted yields (15.5 ± 0.2 and 8.2 ± 0.3 molecules per 100 eV) for the aerated and deaerated Fricke dosimeter when exposed to ^60^Co γ-rays or fast electrons, without additives.

Figure 6a–d illustrate the impact of dose rate, quantified by *N* (the number of proton tracks per pulse; see Section 3.2.3) on the Fricke yields in the radiolysis of aerated and deaerated Fricke-cystamine and Fricke-cysteamine solutions, using low-LET 300 MeV irradiating protons and covering a broad range of cystamine or cysteamine concentrations, from 10^−6^ to 0.1 M. For this study, simulations were performed with *N* values of 1, 10, 50, and 100, where *N* = 100 corresponds to an absorbed dose rate of about 3 × 10^8^ Gy/s under our irradiation conditions [34]. Data for *N* = 1, representing the history of a single proton track, i.e., indicating no dose-rate effects, are utilized as a reference.

In both aerated and deaerated conditions, a consistent trend is observed across all *N* values, mirroring the outcomes depicted in Figure 4a–d, where similar conditions without dose-rate effects but different radiation qualities (LET) are presented. There is a notable and gradual reduction in Fricke yields, especially at lower cystamine or cysteamine concentrations. For instance, at a concentration of 10^−6^ M, where the decrease is most significant, *G*(Fe^3+^) declines from ~15.5 to 13.2 molecules per 100 eV—a reduction of ~2.3 *G*-units—between *N* = 1 and 100 in aerated conditions with cystamine. A similar reduction occurs with cysteamine, where *G*(Fe^3+^) drops from ~15.5 to 13.3 molecules per 100 eV. In deaerated solutions, the decreases in *G*(Fe^3+^) at this concentration are also notable, from 8.1 to 6.9 molecules per 100 eV between *N* = 1 and 100 with cystamine, and about the same with cysteamine.

Similar to the impact of LET, observed in the radiolysis of Fricke-cystamine and Fricke-cysteamine and depicted in Figure 4a–d, the effects of dose rate are predominant in the radioprotection of Fe^2+^ ions at low concentrations across both oxygen-rich and oxygen-depleted environments. Interestingly, as the concentrations of cystamine or cysteamine increase, the reductions in *G*(Fe^3+^) become progressively less marked. This trend indicates that within this range of *N* values, the influence of dose rate wanes in comparison to the effects of cystamine or cysteamine, which increasingly dominate in enhancing radioprotection at higher concentrations.

As highlighted in Section 3.1, high dose rates influence radiolysis yields in a manner akin to high-LET radiation, even though their underlying mechanisms differ. This observed similarity between the effects of dose rate and LET, as noted in [34,36], is a direct consequence of an increase in radical–radical reactions—inter-track reactions across the system as a whole under high dose-rate conditions and intra-track reactions within individual radiation tracks under high-LET conditions. These reactions limit the number of radicals that can avoid combination or recombination, thereby reducing the number of radicals available to react with cystamine or cysteamine in the solution. Consequently, this reduction diminishes the protective efficacy of these compounds. From a radiation-chemical perspective, dose rates therefore mirror the effects of LET, acting as effective radioprotectors.

## 3. Materials and Methods

### 3.1. The Aqueous Ferrous Sulfate (Fricke) Chemical Dosimeter

The ferrous sulfate dosimeter, also known as the Fricke dosimeter, derives its name from Hugo Fricke, who first described its properties between 1927 and 1929 [56,57]. It stands as one of the most thoroughly understood and widely used liquid chemical dosimeters. Its prominence in radiation-chemical work is attributed to its precise and reproducible measurements, along with its linear response to absorbed doses. These characteristics have made it a standard choice in the field [23,63,64,65].

The solution commonly referred to as the ‘standard’ Fricke solution [23,57] comprises 1 mM ferrous sulfate in air-saturated (~2.5 × 10^−4^ M of dissolved O_2_) aqueous 0.4 M sulfuric acid, and has a pH of 0.46. The chemistry of this system primarily involves the oxidation of Fe^2+^ to Fe^3+^ ions, driven by oxidizing species (^•^OH, HO_2_^•^—given the rapid conversion of e^−^_aq_ to H^•^ at low pH and to HO_2_^•^ in the presence of oxygen—and H_2_O_2_) generated through the radiolytic decomposition of (acidic) water [23,57,66]. The main reactions involved in the mechanism for producing Fe^3+^ ions at room temperature are as follows [25,53,68,69,77]:

e^−^_aq_ + H^+^ → H^•^                             *k* = 2.13 × 10^10^ M^−1^ s^−1^ (1)

H^•^ + O_2_ → HO_2_^•^                           *k* = 1.31 × 10^10^ M^−1^ s^−1^ (2)

^•^OH + Fe^2+^ → Fe^3+^ + OH^−^               *k* = 3.4 × 10^8^ M^−1^ s^−1^ (3)

^•^OH + HSO_4_^−^ → H_2_O + SO_4_^•−^          *k* = 1.5 × 10^5^ M^−1^ s^−1^ (4)

SO_4_^•−^ + Fe^2+^ → Fe^3+^ + SO_4_^2−^            *k* = 1.05 × 10^9^ M^−1^ s^−1^ (5)

HO_2_^•^ + Fe^2+^ → Fe^3+^ + HO_2_^−^            *k* = 7.9 × 10^5^ M^−1^ s^−1^ (6)

HO_2_^−^ + H^+^ → H_2_O_2_                        *k* = 5.02 × 10^10^ M^−1^ s^−1^ (7)

H_2_O_2_ + Fe^2+^ → Fe^3+^ + ^•^OH + OH^−^             *k* = 52 M^−1^ s^−1^ (8)

H^•^ + Fe^2+^ (+H^+^) → Fe^3+^ + H_2_              *k* = 1.3 × 10^7^ M^−1^ s^−1^, (9)

where the rate constants (*k*) given here for the reactions between ions are at infinite dilution of ions or zero ionic strength.

Considering all sources of Fe^3+^ ions, the Fricke *G*-value in the presence of O_2_ correlates with the primary yields of radical and molecular species from the radiolysis of water in 0.4 M sulfuric acid, according to the following stoichiometric equation:

*G*(Fe^3+^)_aerated_ = *g*(^•^OH) + 3 *g*(e^−^_aq_ + H^•^) + 2 *g*(H_2_O_2_) + 3 *g*(HO_2_^•^) (10)

where for ^60^Co γ-rays or accelerated electrons, *g*(^•^OH) = 2.90, *g*(e^−^_aq_ + H^•^) = 3.70 is the combined primary yield of e^−^_aq_ and H^•^, *g*(H_2_O_2_) = 0.80, and *g*(HO_2_^•^) = 0.02 [78]. Using these values in Equation (10) yields a *G*(Fe^3+^) value in aerated conditions that aligns closely with the published data for ^60^Co γ-irradiated Fricke solutions at 25 °C, specifically 15.5 ± 0.2 ions/100 eV [23,57,63,64,65,66,67]. This alignment particularly supports the recent measurements by the ‘Ionizing Radiation Standards’ group at the National Research Council of Canada, which reported values of 15.53 and 15.56 ions per 100 eV, with respective uncertainties of ~0.5% and 0.3% [63,64].

In the absence of O_2_, H^•^ cannot react with oxygen and instead serves as an oxidant for Fe^2+^. Consequently, reaction (9) supplants reaction (2), with H^•^ oxidizing only one Fe^2+^ ion, as opposed to three in an aerated solution. Under these conditions, the Fricke *G*-value is determined by the following:

*G*(Fe^3+^)_deaerated_ = *g*(^•^OH) + *g*(e^−^_aq_ + H^•^) + 2 *g*(H_2_O_2_) + 3 *g*(HO_2_^•^) (11)

Reapplying the primary yields of the radical and molecular species given above results in a *G*(Fe^3+^) value that, similar to the aerated medium, closely matches the experimentally observed *G*(Fe^3+^) in deaerated conditions. This value is reported as 8.2 ± 0.3 ions per 100 eV for ^60^Co γ-rays or fast electrons [23,57,66,67].

Equations (10) and (11) demonstrate how the production of Fe^3+^ ions is significantly influenced by factors that affect the primary radical yields, especially the yield of H^•^ atoms. In a 0.4 M H_2_SO_4_ aqueous environment, this yield includes both H^•^ atoms formed directly from radiolysis and those produced indirectly through the protonation of e^−^_aq_. A critical factor here is the LET, which describes the radiation beam’s quality. Numerous studies have consistently shown a decrease in *G*(Fe^3+^) as LET increases [23,37,55,66,67,68,69,70,71,72,73,74]. This reduction correlates with a rise in radical–radical recombination reactions, more prevalent due to the increased local concentration of radicals in the dense tracks of high-LET radiation. With a higher LET, recombination captures more radicals during the expansion of these tracks, thereby enhancing the formation of molecular products [32,33]. Essentially, high-LET radiation restricts the escape of free radicals that would otherwise leave the tracks to oxidize ferrous ions.

Similar to the effects seen with high-LET radiation, high-dose-rate radiation also reduces *G*(Fe^3+^), albeit through distinct mechanisms. At high dose rates, the probability of radical–radical reactions involving H^•^, ^•^OH, and HO_2_^•^ radicals—either singly or in combination—increases throughout the bulk of the solution, driven by intertrack reactions [34,35,36]. This contrasts with high-LET exposures, where this probability intensifies within the confines of individual radiation tracks, underscoring the role of intra-track reactions. This detailed understanding highlights the complex interactions between radiation dose, dose rate, radiation quality (LET), and chemical yields in Fricke dosimetry, demonstrating how these factors jointly influence the measurement outcomes of *G*(Fe^3+^).

### 3.2. Monte Carlo Chemical Modeling of Radiolysis in Fricke-Cystamine and Fricke-Cysteamine Solutions: Chemical Reaction Scheme and Effects of LET and Dose Rate

To examine the radiolysis of Fricke-cystamine and Fricke-cysteamine solutions under both aerated and deaerated conditions at 25 °C, we employed a refined version of our Monte Carlo simulation code, IONLYS-IRT [33,79,80], paired with a recently developed multi-track irradiation model [34]. This model enables proton irradiation at various LETs and dose rates. While the details of these multi-track chemistry simulations are thoroughly documented in earlier publications [34,36,37,53,54,55], we provide a brief summary of their key features here.

#### 3.2.1. The IONLYS-IRT Simulation Code and the Chemical Reaction Scheme

Our ‘IONLYS’ program simulates the initial physical and physicochemical stages of radiation action within a 3D geometric environment, capturing events up to 1 picosecond (ps) in track development. It precisely models, event by event, all fundamental physical interactions associated with energy deposition and the subsequent transformation of locally generated physical products into various initial radical and molecular radiolysis products. These include e^−^_aq_, H^•^, H_2_, ^•^OH, H_2_O_2_, H^+^, OH^−^, HO_2_^•^/O_2_^•−^, O(^1^*D*) and ^•^O^•^(^3^*P*) (oxygen atoms in both singlet ^1^*D* excited state and triplet ^3^*P* ground state, respectively), and O^•−^, among others [33]. This detailed and highly nonhomogeneous spatial distribution of reactants generated by this program serves as the basis for the subsequent chemical stage of the radiolysis process, which begins post 1 ps. In this third stage, the various radiolytic products randomly diffuse from their origin points at rates governed by their diffusion coefficients. They either react with one another or, competitively, with uniformly distributed solutes in the system. Our ‘IRT’ program [80] handles this stage using the “independent reaction times” (IRT) method [81,82,83], a stochastic simulation technique that efficiently calculates reaction times without needing to track the trajectories of each diffusing species. The reliability of this program in providing accurate chemical yields over time has been thoroughly validated under a diverse array of irradiation conditions. This validation comes from comparing its outcomes with those from detailed random flight (or ‘step-by-step’) Monte Carlo simulations, which closely track the trajectories of diffusing reactive species [84]. Furthermore, our IRT program is adept at modeling reactions over prolonged periods of time, when tracks have dissipated and the radiolytic products are uniformly distributed in the solution. This capability is particularly crucial for simulating the radiolysis of the Fricke dosimeter, where Fe^3+^ ions are generated at various time points up to ~200 s (e.g., see [37,53,54,55,68,69,78]).

In our simulation of the radiolysis of Fricke solutions, both aerated and deaerated, with the presence of cystamine (RSSR) or cysteamine (RSH), we adopted the chemical reaction scheme, rate constants, and diffusion coefficients for reactive species in our IRT program as detailed in previous studies [37,53,54,55]. Notably, our reaction model for the aqueous radiation chemistry of cystamine precisely matched the experimental yields of Fe^3+^ observed in Fricke-cystamine solutions irradiated with X- and ^60^Co γ-rays, achieved without the need for adjustable parameters. This accuracy was maintained across a wide range of cystamine concentrations, irrespective of oxygen presence [37,53,54]. The strong agreement between our simulated *G*(Fe^3+^) values and the observed data validates the effectiveness of our reaction scheme in capturing the radiation chemistry of cystamine and, by extension, that of cysteamine, in Fricke solutions. Specifically, we supplemented the Fricke dosimeter reaction scheme by including the 27 chemical reactions found in Table 2 of Meesat et al. [53]. Among these, the reactions critical to the production of ferric ions include the following [85]:

RSSR + e^−^_aq_ → (RSSR)^•−^                                *k* = 4.1 × 10^10^ M^−1^ s^−1^ (12)

RSSR + H^•^ → RS^•^ + RSH                             *k* = 8 × 10^9^ M^−1^ s^−1^ (13)

RSSR + ^•^OH (+H^+^) → (RSSR)^•+^ + H_2_O            *k* = 1.7 × 10^10^ M^−1^ s^−1^ (14)

(RSSR)^•−^ + H^+^ → RS^•^ + RSH                           *k* = 4.2 × 10^9^ M^−1^ s^−1^ (15)

(RSSR)^•+^ + (RSSR)^•+^ → (RSSR)^2+^ + RSSR           *k* = 2.5 × 10^9^ M^−1^ s^−11^ (16)

RS^•^ + RSSR → RSSSR + R^•^                         *k* = 10^6^ M^−1^ s^−1^ (17)

RSH + e^−^_aq_ (+H^+^) → R^•^ + H_2_S                        *k* = 3 × 10^10^ M^−1^ s^−1^ (18)

RSH + H^•^ → RS^•^ + H_2_                                  *k* = 1.8 × 10^9^ M^−1^ s^−1^ (19)

RSH + ^•^OH → RS^•^ + H_2_O                               *k* = 1.7 × 10^10^ M^−1^ s^−1^ (20)

RS^•^ + RSH → RSSR^•−^ + H^+^                            *k* = 3.5 × 10^8^ M^−1^ s^−1^ (21)

R^•^ + RSH → RH + RS^•^                                    *k* = 1.1 × 10^8^ M^−1^ s^−1^ (22)

RS^•^ + RS^•^ → RSSR                                         *k* = 1.5 × 10^9^ M^−1^ s^−1^ (23)

RS^•^ + O_2_ → RSOO^•^                                     *k* = 2 × 10^9^ M^−1^ s^−1^ (24)

RSOO^•^ + RSH → RSO^•^ + RSOH                  *k* = 2 × 10^6^ M^−1^ s^−1^ (25)

R^•^ + O_2_ → ROO^•^                                           *k* = 2 × 10^9^ M^−1^ s^−1^ (26)

Fe^2+^ + RS^•^ (+H^+^) → Fe^3+^ + RSH                       *k* = 2.5 × 10^8^ M^−1^ s^−1^ (27)

Fe^2+^ + (RSSR)^•+^ → Fe^3+^ + RSSR                     *k* = 2 × 10^6^ M^−1^ s^−1^ (28)

Fe^2+^ + RSOO^•^ (+H^+^) → Fe^3+^ + RSOOH             *k* = 10^7^ M^−1^ s^−1^ (29)

Fe^2+^ + ROO^•^ (+H^+^) → Fe^3+^ + ROOH                   *k* = 7.9 × 10^5^ M^−1^ s^−1^ (30)

Fe^2+^ + RSO^•^ (+H^+^) → Fe^3+^ + RSSR                        *k* = 5 × 10^5^ M^−1^ s^−1^, (31)

where the rate constants specified here for reactions involving ions are based on conditions of infinite dilution, where ion–ion interactions are absent. Within the IRT program, we considered the ionic strength of the solutions for all ion reactions, except for the self-recombination of e^−^_aq_, as there is no evidence that ionic strength impacts this reaction rate [86]. The adjustment of these rate constants for ionic strength was carried out following the same method previously employed by Meesat et al. [53].

In this study, under the irradiation conditions used, the concentrations of radiolytic products remained low relative to the background levels of H^+^ (~0.4 M), Fe^2+^ ions (1 mM), O_2_ (~0.25 mM), and cystamine or cysteamine (up to 0.1 M) in the solution. As a result, their reactions could be modeled as pseudo-first order within the IRT program. Additionally, we omitted the ‘direct’ effects of ionizing radiation on the various solutes in the solution. This approximation is deemed reasonable based on the concentrations of H_2_SO_4_, ferrous ions, dissolved O_2_, and either cystamine or cysteamine considered in our study. Finally, the diffusion coefficients for the various species in our IRT simulations of the radiolysis of the Fricke-cystamine and Fricke-cysteamine solutions were taken from existing literature [25,68,69,80]. Since the diffusion coefficient of cystamine in water is not documented, we adopted a value of 2 × 10^−9^ m^2^ s^−1^, similar to that of cysteamine [87]. This value was consistently applied to all derivatives of cystamine and cysteamine as well.

#### 3.2.2. Assessing the Effect of LET with Protons of Various Energies

As outlined in Section 1.1, at low LET, radiation initially forms distinct, spherical spurs that evolve independently with minimal reactant overlap, primarily resulting in radical production. With increasing LET, these spurs begin to merge due to the diffusion of reactive species, forming a dense, continuous, cylindrical column along the radiation track [29,30,31]. This proximity increases the probability of radical–radical combination and recombination reactions during the track stage of radiolysis. Unlike low-LET radiation, which produces a high yield of radicals, densely ionizing radiation enhances the production of molecular products such as H_2_ and H_2_O_2_, and facilitates water reformation [32,33].

In this study, we quantitatively assessed the effect of LET on the formation of Fe^3+^ ions during the radiolysis of Fricke-cystamine and Fricke-cysteamine. We employed protons at four different energies as radiation sources—0.15, 6, 70, and 300 MeV—with respective mean LET values at 25 °C of ~72, 6.8, 0.9, and 0.3 keV/μm [88,89].

#### 3.2.3. Modeling Dose-Rate Effects Using the “Instantaneous Pulse” (Dirac) Model

In our study, we employed a multi-track irradiation model recently developed in our laboratory to explore the influence of dose rate on the radiolysis of water and Fricke-cystamine solutions by incident protons [34,36,54,90] and swift carbon ions [91]. Briefly, the model involves randomly irradiating water with single pulses of *N* monoenergetic protons that simultaneously impact the water perpendicularly over the surface of a circular area with radius R_o_, as depicted in Figure 1 of Alanazi et al. [34]. This approach is referred to as the “instantaneous pulse” or Dirac model, where the pulse duration is effectively zero [65], meaning that all chemical species are generated instantaneously. In this context, the absorbed dose per pulse is the only parameter of interest [65].

The rectilinear trajectories of the fast protons enable the definition of a cylindrical geometry for the beam at entry, with all proton tracks parallel to the cylinder’s axis. This irradiated cylinder, surrounded by non-irradiated bulk water, allows the initially formed radiolytic species to diffuse throughout the bulk water, which is effectively infinite. In this particular setup, the incident proton “fluence” (the number of impacting protons per unit area) is calculated as *N*/π*R*_o_^2^. By adjusting *N*, the number of protons per pulse, we directly examine the effect of dose rate on our system. In the present study, *N* ranges from 1 to 100. Specifically, data for *N* = 1, which represent conditions with no dose-rate effects, serve as a reference point for comparison.

Based on our previous calibration of *N* in terms of dose rate (see Figure 3b in Alanazi et al. [34]), *N* = 100 corresponds to an absorbed dose rate of ~3 × 10^8^ Gy/s under our conditions of irradiation. Finally, we define time zero as the moment when the *N* incident protons reach the front of the cylinder.

## 4. Conclusions

In this study, we compared the radical-capturing abilities of cystamine (RSSR), a disulfide, and cysteamine (RSH), its thiol monomer, from a radiation-chemical perspective. Using our Monte Carlo multi-track chemistry simulation code, IONLYS-IRT, we specifically examined how these compounds interact with primary chemical species produced during the radiolysis of the ferrous sulfate (Fricke) dosimeter under conditions of high-LET and high-dose-rate irradiation. To assess the radioprotective or antioxidant effectiveness of these compounds, we employed the radiolytic oxidation of Fe^2+^ to Fe^3+^ ions in Fricke-cystamine and Fricke-cysteamine solutions. Our aim was to investigate the molecular mechanisms of radioprotection provided by these compounds when exposed to proton irradiation with initial energies ranging from 300 to 0.15 MeV, corresponding to LET values from ~0.3 to 72 keV/μm. We examined their effectiveness across various dose rates and in both aerated and deaerated environments.

Overall, the decrease in *G*(Fe^3+^) observed in the presence of cystamine or cysteamine is greatly influenced by both the irradiation conditions and the oxygenation levels of the environment. Importantly, two synergistic radioprotective mechanisms have been identified: one is driven by the chemical activity of cystamine or cysteamine in the solutions, and the other arises from the inherent effects of LET or the dose rate, reflecting the physical characteristics of the radiation. Specifically, our findings underscore that the protective benefits of cystamine and cysteamine stem largely from their ability to neutralize the primary products of water radiolysis, highlighting their robust antioxidant properties. The presence or absence of oxygen also critically influences the radioprotective effectiveness of these compounds. In aerated conditions, the oxidation of Fe^2+^ to Fe^3+^ primarily occurs through reactions with HO_2_^•^, H_2_O_2_, and the radical species RS^•^ and RSSR^•+^. In contrast, under deaerated conditions, while the reactions are largely similar, a key distinction arises because H^•^ atoms do not react with oxygen.

The findings of this study offer significant predictive insights. For instance, under low-LET irradiation conditions, cystamine is notably more effective than cysteamine as a radioprotector in aerated environments—which simulate healthy tissues—at concentrations up to ~1 mM. At higher concentrations within these low LET conditions, both cystamine and cysteamine display strong radioprotective qualities, though cystamine maintains a slight advantage. In contrast, at high LET, cysteamine demonstrates a significantly greater ability than cystamine to mitigate the harmful effects of ionizing radiation at concentrations exceeding ~1 mM, where *G*(Fe^3+^) experiences a notable reduction, attributable to the unique chemical properties of cysteamine.

In deaerated environments that mimic hypoxic (oxygen-depleted) tumor tissues, cysteamine shows negligible radioprotective effects on Fe^2+^ ions under low-LET conditions, irrespective of concentration. Therefore, among the two compounds studied, cysteamine is optimally effective as a radioprotector for healthy (oxygenated) tissue at concentrations above 1 mM during conventional, low-LET radiotherapy, where the primary aim is to shield healthy tissues from radiation damage while specifically targeting tumor cells. On the other hand, cystamine reliably offers robust protection in both oxygen-rich and oxygen-poor conditions, underscoring its broad applicability across various environmental contexts.

Finally, we also investigated the effects of high dose rates on the radioprotective and antioxidant properties of cystamine and cysteamine. Our primary observation is the notable similarity between the impacts of dose rate and LET on radiolysis yields, despite their distinct underlying mechanisms. This correlation closely matches findings from the literature, which examine how LET and dose rate impact the chemistry and yields in the radiolysis of both aerated and deaerated Fricke dosimeters, whether or not additives are present.

Assuming that the distinct protective roles of cystamine and cysteamine observed in this study are applicable to biological systems, and setting aside potential in vivo toxicity, there is a strong justification for incorporating these compounds into clinical practice as adjuncts to radiation therapy, particularly with advanced techniques like FLASH-RT. Their inclusion could potentially mitigate the side effects of radiation exposure, thereby enhancing patient quality of life and improving treatment outcomes. However, the varied effectiveness of these compounds under different conditions highlights the need for personalized approaches in their application, taking into consideration the specific characteristics of the tumor and the surrounding healthy tissues.

Furthermore, this study emphasizes the vital importance of continued research and development in the field of radioprotective agents. The quest for compounds that can provide effective protection against the harmful effects of radiation—while minimizing side effects and maintaining therapeutic efficacy—poses a significant challenge. The insights gained from this comparative analysis of cystamine and cysteamine pave the way for future research, prompting the exploration of novel compounds and their combinations to augment the radioprotective toolkit available for clinical application.

This study not only enhances our understanding of the radioprotective and antioxidant mechanisms of cystamine and cysteamine but also highlights their potential to improve the therapeutic ratio of cancer treatments involving radiation. The detailed insights into their efficacy across various environmental conditions provide valuable guidance for their clinical use, underscoring the need for a personalized and strategic deployment of radioprotective agents to optimize cancer treatment protocols. As we further explore the frontiers of radiation therapy and strive to maximize its therapeutic potential, the role of radioprotective agents like cystamine and cysteamine will undoubtedly be pivotal in shaping future treatment approaches.

Advancements in this field also include the integration of nanotechnology with synthetic radioprotectors to improve delivery and specificity, which could potentially reduce side effects and enhance protective efficacy. Additionally, investigating natural compounds with radioprotective properties introduces new possibilities for enhancing radioprotective strategies, merging traditional chemical approaches with biotechnological innovations. This convergence marks a promising new frontier in the fight against radiation-induced cellular damage.

## Data Availability

The original contributions presented in the study are included in the article, further inquiries can be directed to the corresponding author.
